# BRANT: A Versatile and Extendable Resting-State fMRI Toolkit

**DOI:** 10.3389/fninf.2018.00052

**Published:** 2018-09-03

**Authors:** Kaibin Xu, Yong Liu, Yafeng Zhan, Jiaji Ren, Tianzi Jiang

**Affiliations:** ^1^Brainnetome Center, Institute of Automation, Chinese Academy of Sciences, Beijing, China; ^2^National Laboratory of Pattern Recognition, Institute of Automation, Chinese Academy of Sciences, Beijing, China; ^3^Sino-Danish Center for Education and Research, Beijing, China; ^4^Sino-Danish College, University of Chinese Academy of Sciences, Beijing, China; ^5^School of Artificial Intelligence, University of Chinese Academy of Sciences, Beijing, China; ^6^CAS Center for Excellence in Brain Science, Institute of Automation, Chinese Academy of Sciences, Beijing, China; ^7^School of Biomedical Engineering, Southern Medical University, Guangzhou, China; ^8^Key Laboratory for NeuroInformation of Ministry of Education, School of Life Science and Technology, University of Electronic Science and Technology of China, Chengdu, China; ^9^Queensland Brain Institute, University of Queensland, Brisbane, QLD, Australia

**Keywords:** BRANT, resting-state fMRI, code-generated GUI, preprocessing, visualization

## Abstract

Data processing toolboxes for resting-state functional MRI (rs-fMRI) have provided us with a variety of functions and user friendly graphic user interfaces (GUIs). However, many toolboxes only cover a certain range of functions, and use exclusively designed GUIs. To facilitate data processing and alleviate the burden of manually drawing GUIs, we have developed a versatile and extendable MATLAB-based toolbox, BRANT (BRAinNetome fmri Toolkit), with a wide range of rs-fMRI data processing functions and code-generated GUIs. During the implementation, we have also empowered the toolbox with parallel computing techniques, efficient file handling methods for compressed file format, and one-line scripting. In BRANT, users can find rs-fMRI batch processing functions for preprocessing, brain spontaneous activity analysis, functional connectivity analysis, complex network analysis, statistical analysis, and results visualization, while developers can quickly publish scripts with code-generated GUIs.

## Introduction

Resting-state fMRI (rs-fMRI) has been studied intensively not only as a reference for task fMRI but also as a technique to detect intrinsically synchronized signals among brain areas (Fox et al., 2005). Rs-fMRI signals are reconstructed from blood oxygen level-dependent (BOLD) signals, which fluctuate with the ratio of oxygenated to deoxygenated hemoglobin in the vicinity (Ogawa et al., 1990). To study rs-fMRI signals, a great number of methods and software such as Statistical Parametric Mapping (SPM) (Ashburner, 2012), FMRIB Software Library (FSL) (Smith et al., 2004), Analysis of Functional NeuroImages (AFNI) (Cox, 1996, 2012) have been developed to provide users executable software, scripts and graphic user interfaces (GUIs) for data preprocessing, metrics calculation, statistical analysis, and results visualization. And to facilitate data preprocessing, software packages such as the 1000 Functional Connectomes Project scripts (http://fcon_1000.projects.nitrc.org/), the minimal preprocessing pipelines (Glasser et al., 2013), the toolbox for Data Processing & Analysis for Brain Imaging (DPABI) (Yan et al., 2016), CONN (Whitfield-Gabrieli and Nieto-Castanon, 2012) and the GRaph thEoreTical Network Analysis (GRETNA) toolbox (Wang et al., 2015), have integrated functions of SPM, FSL and AFNI into their pipelines for batch preprocessing. After preprocessing steps such as temporal corrections, spatial alignment, space registrations, and noise removal, rs-fMRI signals can be further processed by measuring temporal, frequential, and spatial properties. Frequently used methods can be coarsely categorized as, but not restricted to, functional connectivity-based methods, complex network-based methods, and voxel-wise spontaneous activity-based methods. Functional connectivity-based methods are used to calculate pair-wise properties such as temporal dependency (Biswal et al., 1995), coherence (Sun et al., 2004), and maximum information coefficient (Reshef et al., 2011), between regions of interest (ROIs) or between seed ROI and whole-brain voxels. Given a connectivity matrix, complex network-based methods can be used to capture the topological properties of the entire network, as well as its components (subgraphs, nodes, and edges). With increased computing capability, voxel-wise methods have been implemented to describe temporal, frequently and spatial profiles of each voxel's time series (Zang et al., 2004, 2007; Tomasi and Volkow, 2010). To test hypothesis, statistical analysis methods are used, and to present results, visualization methods are implemented as displaying slices, projecting volumes, rendering surfaces, plotting topological connectivity, and etc. Most of the above mentioned methods are implemented in SPM (Ashburner, 2012), FSL (Smith et al., 2004), AFNI (Cox, 1996, 2012), Network Based Statistic (NBS) (Zalesky et al., 2010), Brain Connectivity Toolbox (BCT) (Rubinov and Sporns, 2010), CONN (Whitfield-Gabrieli and Nieto-Castanon, 2012), REST (Song et al., 2011), DPABI (Yan et al., 2016), GRETNA (Wang et al., 2015), xjView (http://www.alivelearn.net/xjview), Caret (Van Essen et al., 2001), Connectome Workbench (Marcus et al., 2013), BrainNet Viewer (Xia et al., 2013), and MRIcron/Mricro (Rorden and Brett, 2000).

Different software packages have provided us with efficient batch processing pipelines which can be run by clicking on GUIs instead of writing scripts. However, in most of the above-mentioned software, the number of functions is fixed and GUIs are exclusively designed. During data processing, users would need to turn to multiple software packages and sometimes write scripts for batch processing. To facilitate data processing, we have written a versatile and extendable MATLAB-based toolbox, BRANT (BRAinNetome fmri Toolkit, https://brant.brainnetome.org), which integrates batch processing pipelines for rs-fMRI data preprocessing, voxel-wise spontaneous activity analysis, functional connectivity analysis, complex network analysis, as well as statistical analysis and results visualization. In BRANT, we designed the GUIs to be code-generated, which requires only a few lines of MATLAB code to add new functions. We have also optimized computing-related scripts by matrix operations, parallel computing techniques and multithreading, and optimized file-handling-related scripts by MATLAB-C++ compiled ^*^.mex executives that load and save gzipped files directly.

In the following paragraphs, we at first describe the functions of BRANT in theory and formula, then we demonstrate the examples of code-generated GUI design in the cases of Regional Homogeneity (ReHo) and two third-party software. In the end, we compare BRANT with three other frequently used MATLAB-based rs-fMRI data processing software and discuss the advantages and limitations of our toolbox.

## Materials and methods

### Overview

**Figure 1:**
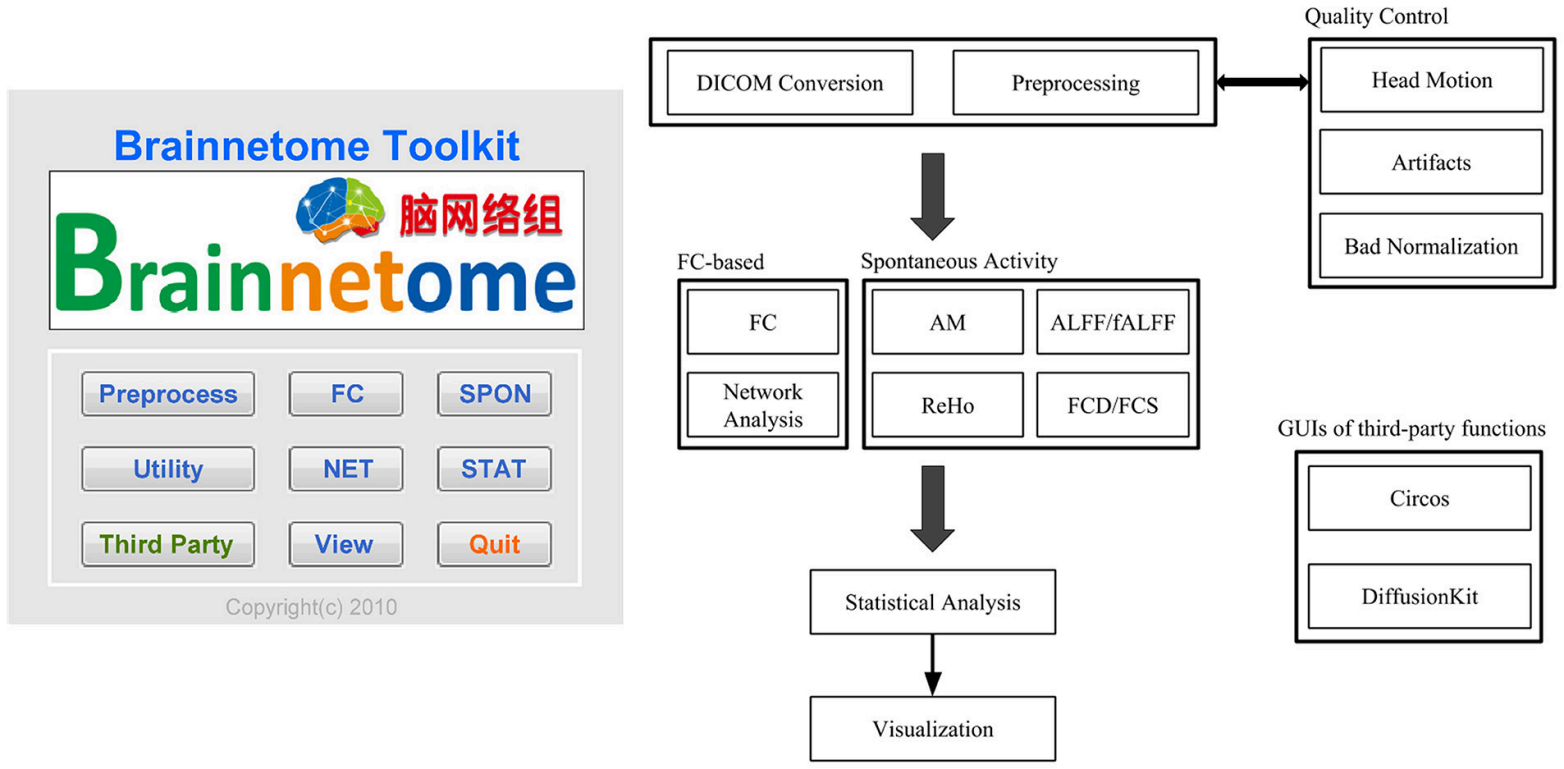
Main interface and workflow of BRANT.

The main part of BRANT is composed of MATLAB scripts and code-generated GUIs. To simplify the input process, most functions are initialized with default settings, and users will only need to specify several necessary parameters, with free access to all. Functions of BRANT are arranged into 7 modules, which are preprocessing, functional connectivity (FC), spontaneous activity (SPON), complex network analysis (NET), statistics (STAT), visualization (View), and utilities (Figure 1).

### Preprocessing

Raw data collected from MR scanners are formatted as DICOM (Digital Imaging and Communications in Medicine) files, and each file stores the intensity of one volume or one slice. To process data more efficiently, DICOM files are firstly converted to a single 4D NIfTI (Neuroimaging Informatics Technology Initiative) image by *dcm2nii* in MRIcron (Rorden and Brett, 2000), which has been well-tested and is known within the community as being stable and fast (Jenkinson et al., 2012). For converted data, visual inspection is recommended to censor data with low quality (artifacts and distortions). Qualified data can be further processed in the preprocessing pipeline. Since SPM (Ashburner, 2012) has implemented preprocessing functions that are widely used by most MATLAB-based toolboxes, we follow the convention and use SPM's built-in scripts for slice timing correction, realignment, co-registration, spatial normalization and smoothing, along with our *Denoise* scripts for covariates regression and band-pass filtering in the batch preprocessing pipeline.

In *Denoise*, time series of one subject's 4D volume is extracted and reshaped to a temporal-spatial 2D matrix as the input of a multiple regression model and a square-wave filter. In the regression model, physiological noises and head motion effects addressed by Friston et al. (1996) and Power et al. (2014) are used as noise regressors, which include linear trend, mean time series extracted from tissue masks (white matter WM, cerebrospinal fluid CSF, and global signal GS, denoted as *T*) and estimated head-motion parameters (*R*, 3 translational and 3 rotational estimates output by *Realign*), variant forms of *T* and *R*, as well as all the squared forms of the above regressors. The variant forms include the first derivatives with zero padding and 1-frame lagged time series with zero padding. Each noise regressor is optional, and can be packed into the different regression models suggested in (Ciric et al., 2017). Since whether to regress out global signal is controversial (Fox et al., 2009; Murphy et al., 2009; Hahamy et al., 2014), an option is offered to perform either or both operations. Before the regression model, an optional strategy called *scrubbing* is implemented to censor large motion frames with a threshold of the FD (framewise displacement) (Power et al., 2012, 2014).

$$\begin{array}{l} T=[WM,\;CSF,\;GS]\\ R=[x,\;y,\;z,pitch,yaw,\;roll]\\ X=[ones,\;trends,T,T^{2},T\prime ,T\prime^{2},T_{t-1},T_{t-1}^{2},R,R^{2},R\prime ,R\prime^{2},R_{t-1},R_{t-1}^{2}]\\ y=\beta X+\epsilon \end{array}$$

*T*: mean tissue time series. *R*: estimated head motion of each temporal frame. *ones*: a column of 1 representing the regressor of intercept. *X*: matrix of regressors with intercept. *trends*: Linear and quadratic trends. Prime (′): first derivatives against time with zero padding. t-1: 1-frame lagged signal with zero padding. *y*: time series of each voxel. β: estimated weights of each regressor. ϵ: residual and output of the regression model

In *Filter*, each voxel's time series is transformed into frequency domain using fast Fourier transform (FFT). The frequency-domain signals are then filtered by an ideal band-pass filter and transformed back to time domain using inverse FFT.

### Functional connectivity (FC)

Functional connectivity is calculated as the temporal correlation between pairs of time series extracted from ROIs or voxels. In BRANT, three methods of preparing ROIs are provided, including drawing spheres/cubes from coordinates, extracting ROIs from an atlas and merging separate ROI files into one number-tagged template.

#### Draw regions of interest (Draw ROIs)

*Draw ROIs* is implemented as automatically drawing spheres or cubes with ROI coordinates and a header reference 3D image. The ROI coordinates and labels are sorted in a ^*^.csv table for output indexing purpose, while the header reference image is used to define the output image properties such as the bounding box, originator, orientation, inclusive mask, and voxel size.

#### Merge/extract ROIs

Given a number-tagged atlas such as the Brainnetome Atlas (Fan et al., 2016), a subset of ROIs indexed by integers can be extracted and exported to one 3D image. Conversely, given separated ROI files, the current function can also merge them into one combined atlas-like ROI file, with ROI labels stored in a ^*^.csv table. More atlases will be added in the future.

#### ROI signal calculation

With a predefined atlas-like ROI file and a descriptive number-label table, the current function can extract mean time series from ROIs and voxels, and calculate Pearson's correlation as well as its Fisher-z transform.

$$\begin{array}{l} rho\left(x,\;y\right)=\;\frac{cov\left(x,\;y\right)}{std\left(x\right)*\;std\left(y\right)}\\ {rho\left(x,\;y\right)}_{z}=0.5\;*\log\left(\frac{1+rho\left(x,\;y\right)}{1-rho\left(x,\;y\right)}\right) \end{array}$$

*rho*(*x, y*): Pearson's correlation of two time series, *x* and *y. cov*(*x, y*): covariance of *x* and *y. std*(*x*): standard deviation of x. *rho*(*x, y*)_*z*_: Fisher-z transform of the Pearson's correlation.

An option is provided to calculate partial correlation between each pair of ROIs, with mean signals of other ROIs as covariates.

### Spontaneous activity (SPON)

Voxel-wise metrics of time series implemented in the current module include amplitude of time series (AM), (fractional) amplitude of low frequency fluctuation (ALFF/fALFF), regional homogeneity (ReHo), functional connectivity density (FCD), and functional connectivity strength (FCS). Two intensity normalization methods are implemented as described in Wu et al. (2009), Hoptman et al. (2010), and Xu et al. (2015) for the above metrics.

$$\begin{array}{l} vm_{i}=\frac{v_{i}}{mean\left(V_{brain\;mask}\right)}\\ vz_{i}=\frac{v_{i}-mean\left(V_{brain\;mask}\right)}{std\left(V_{brain\;mask}\right)} \end{array}$$

*vm*_*i*_: mean normalized intensity of voxel *i. vz*_*i*_: z-score intensity of voxel *i. V*_*brain mask*_: set of intensities of voxels within the brain mask.

#### Amplitude of time series (AM)

AM is calculated as the average amplitude and the standard deviation of the mean-subtracted time series (Liu et al., 2014). The AM represents the strength of time series' temporal fluctuation, which is similar to the ALFF/fALFF.

$$\begin{array}{l} {\text{AM}}_{mean}=\frac{1}{N}\overset{N}{\underset{j=1}{\sum }}|x\left(j\right)-\bar{x}|\\ {\text{AM}}_{std}=\frac{1}{N-1}\overset{N}{\underset{j=1}{\sum }}{\left(x\left(j\right)-\bar{x}\right)}^{2} \end{array}$$

*N*: length of time series. *x*: mean of time series. *std*: corrected sample standard deviation.

#### (Fractional) amplitude of low frequency fluctuation (ALFF/fALFF)

ALFF is calculated as the amplitude of the time series in a certain frequency band (Zang et al., 2007), which is the averaged square root of the power spectral density of the filtered time series. To increase the stability of ALFF across subjects, fALFF was proposed as calculating the fraction of a certain frequency band against the whole available frequency band (Zou et al., 2008).

$$\begin{array}{l} \text{ALFF}=\frac{1}{N}\overset{N}{\underset{i=1}{\sum }}\left(\sqrt{\frac{1}{L}\;*F\;*\;conj\left(F\right)}\right) \end{array}$$

*F*: FFT of the filtered time series. *N*: number of frequency samples within the frequency band. *L*: length of time series. *conj*(*F*): the complex conjugate of the elements of *F*.

#### Regional homogeneity (ReHo)

ReHo is calculated as the Kendall's coefficient of concordance (KCC) among a seed voxel and its neighbor voxels, which indicates the degree of spontaneous activity in the seed voxel's vicinity (Zang et al., 2004). Voxels of higher intensity in ReHo maps indicate greater similarity among neighboring voxels' time series.

$$\begin{array}{l} \text{W}=\;\frac{\sum {\left(R_{i}\right)}^{2}-n{\left(\bar{R}\right)}^{2}}{\frac{1}{12}K^{2}\left(n^{3}-n\right)} \end{array}$$

*W*: Kendall's W. *K*: number of neighbor voxels (7, 19 or 27). *R*_*i*_: sum of all *K* voxels' rank at frame *i. n*: length of time series, or the number of frames. *R*: average *R*_*i*_ across frames.

#### Functional connectivity density and functional connectivity strength (FCD/FCS)

A region growing algorithm was carried out by Tomasi and Volkow (2010) to measure the local degree of each voxel under a certain threshold of Pearson's correlation. FCD in BRANT has been implemented to calculate the local FCD (lFCD), the global FCD (gFCD), and the long-range FCD (lrFCD) at one time. The lFCD of each voxel represents the number of spatially connected voxels defined by the region growing algorithm (Tomasi and Volkow, 2010), while the gFCD, which is also referred to as the voxel-wise degree centrality (Craddock and Clark, 2016), represents the number of voxels that have higher-than-threshold correlation with the seed voxel. The lrFCD is calculated as the gFCD subtracted by the lFCD (Qin et al., 2014).

The original description of the gFCD includes whole-brain voxel-wise correlation, which is time consuming for traditional CPU implementation. In BRANT, we first calculate the gFCD in parallel with OPENCL (https://www.khronos.org/opencl/), and meanwhile keep in memory the thresholded voxel-wise FC matrix in bits, and then carry on the region grow method for each voxel on multiple CPU threads for lFCD. Since the binarized correlation matrices are stored in bits, the required memory is 1 / (4bytes ^*^ 8bits/byte) of the *float* data type, which makes the program run smoothly on a 4GB-memory laptop with a 1GB-memory discrete GPU for preprocessed rs-fMRI 4D images with ~50,000 voxels, ~230 timepoints. The computing time can vary with the distribution of connectivity strength, that when the mean connectivity strength rises, there are more binarized connections as well as more time spent on the region grow method. In our experience, for rs-fMRI image with ~230 timepoints, ~50,000 voxels (3^*^3^*^3 mm^3^ spatial resolution), and the program can finish within 2–5 min. By default, the definition of neighbor type is set as vertex-connected.

Functional connectivity strength (FCS) measures the amount of information a node receives across whole graph or within a distance (Jiang et al., 2004; He et al., 2009; Wang et al., 2014). Similar to FCD, the voxel-wise Pearson's correlation coefficients are firstly calculated in parallel and then Fisher-z transformed to improve normality. For each voxel, the FCS is calculated as the sum of connectivity that exceeds a given threshold divided by the number of voxels.

### Complex network analysis (NET)

Network metrics depict the properties of information flow among predefined nodes. Regarding brain networks, ROIs are defined as nodes and the connections between pairs of ROIs are defined as edges. In the current module, connectivity matrices are firstly thresholded by intensity or sparsity to weighted or binary networks. Then, available complex network metrics including shortest path length, clustering coefficient, small-worldness, global and local efficiency, betweenness centrality, weighted and binary degree centrality, neighbor degree centrality, assortative mixing, resilience, transitivity, fault tolerance, vulnerability are calculated. Detailed network properties have been described by Rubinov and Sporns (2010). For group comparisons under a vector of thresholds, Student's *t*-tests are provided.

### Statistics (STAT)

The current module provides Student's *t*-tests for sample mean comparisons and several methods for image-based meta-analysis (IBMA) (Salimi-Khorshidi et al., 2009). For multi-comparison correction, we use the Benjamini-Hochberg and the Benjamini-Yekutieli procedures to control the false discovery rate (FDR) of dependent and independent cases (Benjamini and Hochberg, 1995; Benjamini and Yekutieli, 2001), and the Bonferroni procedure to control the familywise error rate (FWER).

#### Image-based meta-analysis (IBMA)

With statistical maps of different datasets tested using same analysis pipeline, and the demography of each sample, users can perform meta-analysis to merge the multisite statistics using image-based or matrix-based meta-analysis (Salimi-Khorshidi et al., 2009). We have implemented Stouffer's z-score method (Stouffer et al., 1949), Fisher's method (Fisher, 1925), fixed/mixed effects model (Konstantopoulos, 2006; Hedges, 2016), Worsley and Friston's method (Worsley and Friston, 2000), and Nichols' method (Nichols et al., 2005). Usage descriptions and comparisons have been reviewed in Lazar et al. (2002) and Salimi-Khorshidi et al. (2009).

### Visualization (view)

**Figure 2:**
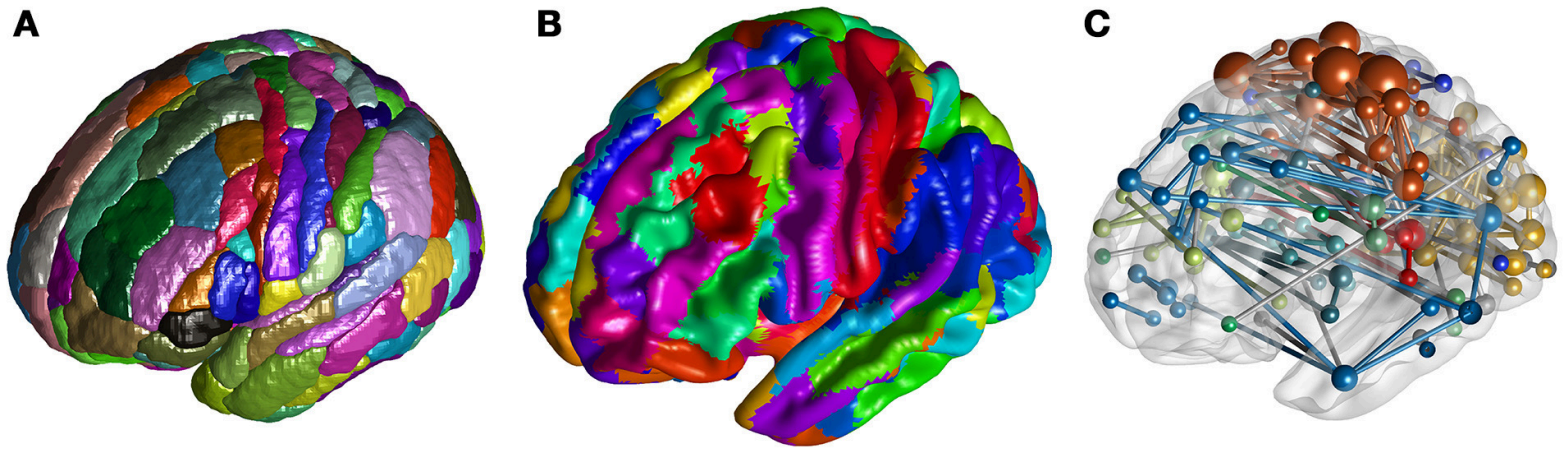
**(A)** Rendered clusters of the Brainnetome Atlas in *ROI Mapping*. **(B)** Projection of the Brainnetome Atlas on a brain surface in *Surface Mapping*. **(C)** *Network Visualization* for a random network.

To visualize voxel intensities, we have implemented the *ROI Mapping* to extract and render the surface of 3D clusters (Figure 2A), the *Surface Mapping* to project voxel intensity to vertices on a surface (Figure 2B). To visualize ROI-ROI connectivity, *Network Visualization* is implemented to draw spheres and rods within a rendered brain surface, to present nodes and edges of the input network (Figure 2C).

#### ROI mapping

When visualizing ROIs from an atlas or clusters from a user-defined 3D volume (e.g., clusters with significant difference between sample means), we can use the current function to extract and shade the surface of each number-tagged ROI/cluster in random or user defined colors (Figure 2A). The ROIs/clusters of the input 3D image should be tagged with positive-integers. With an additional input of a reference ^*^.csv table containing number-label pairs (as described in *Utilities-DICOM Convert*), we can further parse the labels of each shaded ROI/cluster and present them in a legend.

#### Surface mapping

Besides shading each ROI/cluster, we can also project the voxel intensities to the surface. By default, we use a rendered human brain surface constructed from vertices and triangular faces loaded from a pregenerated file (Figure 2B). To draw another surface, users can input a binarized 3D mask, with which BRANT can extract vertices and faces and render a new surface. When projecting a 3D volume to surface, the vertices on the surface are shaded as the intensity of the nearest voxel, while the material of the surface, the colormaps of positive and negative intensities, the lighting and shading algorithms can be adjusted.

#### Network visualization

Using a ^*^.txt file storing symmetric connectivity matrix and a ^*^.csv table with nodal information (such as coordinate, label, module, and color) as input, we can draw spheres and rods to visualize nodes and edges (Figure 2C). An online description of the table and drawing options are provided at https://brant.brainnetome.org.

### Utilities

We have added several frequently used functions in this module to facilitate DICOM image conversion, the process of quality control, ROI coordinates extraction and 2D/3D signal extraction.

#### DICOM convert

Since in practice raw MRI data exported from an MR scanner consists of a large number of DICOM images storing slices and volumes of different sequences, by convention we convert the DICOM images to packed 3D or 4D NIFTI images before all processing steps. In BRANT, we use the *dcm2nii* from MRIcron/MRIcro (Rorden and Brett, 2000) to convert DICOM files into 4D NIfTI images by default and use wildcard characters to locate rs-fMRI image files. For the matched images, the First *N timepoints removal* is used to remove the first N frames that could be influenced by large motion or the instability of magnetic field.

#### Head motion estimation

Head motion has been found having an impact on rs-fMRI signals (Van Dijk et al., 2012). In preprocessing, six head motion parameters of (x-, y-, z-) translations and (pitch-, yaw-, roll-) rotations estimated during realignment are used as the inputs of the current function. By default, the current function outputs the maximum absolute translation and rotation between frames as the exclusion criterion of large-motion subject. Additionally, the mean head displacement (the root-mean-square of translation parameters), the maximum head displacement, the number of micro displacement (>0.1 mm), the mean absolute Euler angle of rotation (Van Dijk et al., 2012), the framewise displacement (FD) (Power et al., 2012) and the number of frames with FD > 0.5 mm, are also exported to provide more subject exclusion criteria.

#### Visual check

The current function provides batch operations to visually inspect artifacts and normalization quality, by calling *Display* from SPM. We've added keyboard operations to the *Display* figure that users can press up/down to switch fMRI volumes of one subject and press left/right to switch subjects. Before running the frame-by-frame inspection, the current function exports screenshots of selected slices overlaid by a semi-transparent brain mask for a glimpse of the overall image quality.

#### Temporal signal to noise ratio (TSNR)

Influenced by the magnetic field inhomogeneity at air-tissue interfaces, rs-fMRI signals at orbitofrontal and temporal medial and polar areas suffer from a certain degree of distortions and signal loss. To exclude spurious voxels, we use the thresholded voxel-wise TSNR, which is calculated as the average intensity of time series divided by the standard deviation (Tomasi and Volkow, 2010; Yeo et al., 2011; Welvaert and Rosseel, 2013), to generate subject-level or group-level whole-brain mask.

#### ROI coordinates

To visualize the topological structure of network connections, ROI coordinates are expected as the centers of spheres. In the current function, coordinate of each number-tagged ROI is calculated as the center of mass with equal weights and then exported to a ^*^.csv table.

#### Extract signals from 3D volume and 2D matrix

Given a 2D binary mask or a 3D number-tagged ROI volume, elements of matrix or mean intensity within each ROI can be extracted and exported to a table with cell value arranged as subjects (rows) x extracted signals (columns).

### Code-generated GUIs and embedded third-party software

GUI panels of BRANT are designed as combinations of code-generated GUI elements that receive and check user inputs. For example, in *ReHo*, the following inputs are expected to locate preprocessed rs-fMRI images in each input directory:

- The directory of each subject's preprocessed data;
- Wildcard characters to locate filename in each directory, such as ‘f^*^.nii’;
- A numeric index to extract one unique string from each subject's directory. For example, the index should be 2 for ‘d:\data\subj001\fmri’, and 1 for ‘d:\data\fmri\subj001’ to parse the “subj001” for output purpose;
- A checkbox indicating whether the input NIFTI files in each directory are multiple 3D files or one 4D file.
- The full path of a 3D mask to restrict the calculation within the brain.

Since most post-processing functions in BRANT require the above inputs, the code-generating process has been simplified as adding one line to a *cell* array (the variable ‘ui_structs’) in ‘brant_postprocess_defaults.m’.

{'sub_gui', 'disp_dirs_nii_mask'}, 'input_nifti', {{'filetype', 'f^*^.nii'}, {'nm_pos', 1}},”;

Items in the first *cell* indicate there is a combination of GUIs named 'disp_dirs_nii_mask' to be parsed in the ‘sub_gui’ *dictionary*. Then, two *fields* of “input_nifti” will be initialized with the third *cell* “{{'filetype', 'f^*^.nii'}, {'nm_pos', 1}}” to change the default wildcard characters of *filetype* to “f^*^.nii” and the default numeric index of output string to 1. For the rest of the example *ReHo* GUI generation, such as neighbor type, normalize options, smooth options and output directory, BRANT uses a similar procedure as above. In the end, we add the ‘process_fun = @brant_reho;’ to specify ‘brant_reho.m’ as the data processing script for the GUI parameters collected by the button labeled with ‘run’. See *case* ‘reho’ in the script ‘brant_postprocess_defaults.m’ for more details. When generating the *ReHo* panel, BRANT at first collects all above GUI parts and default settings, and then breaks each input into basic MATLAB GUI elements (e.g., pushbutton, text, checkbox, pop-up menu) and draws them on the ‘ReHo’ panel along with preset callback functions.

In the following two sections, we introduce two instances of packing batch processing terminal scripts into GUI panels.

#### Circos

**Figure 3:**
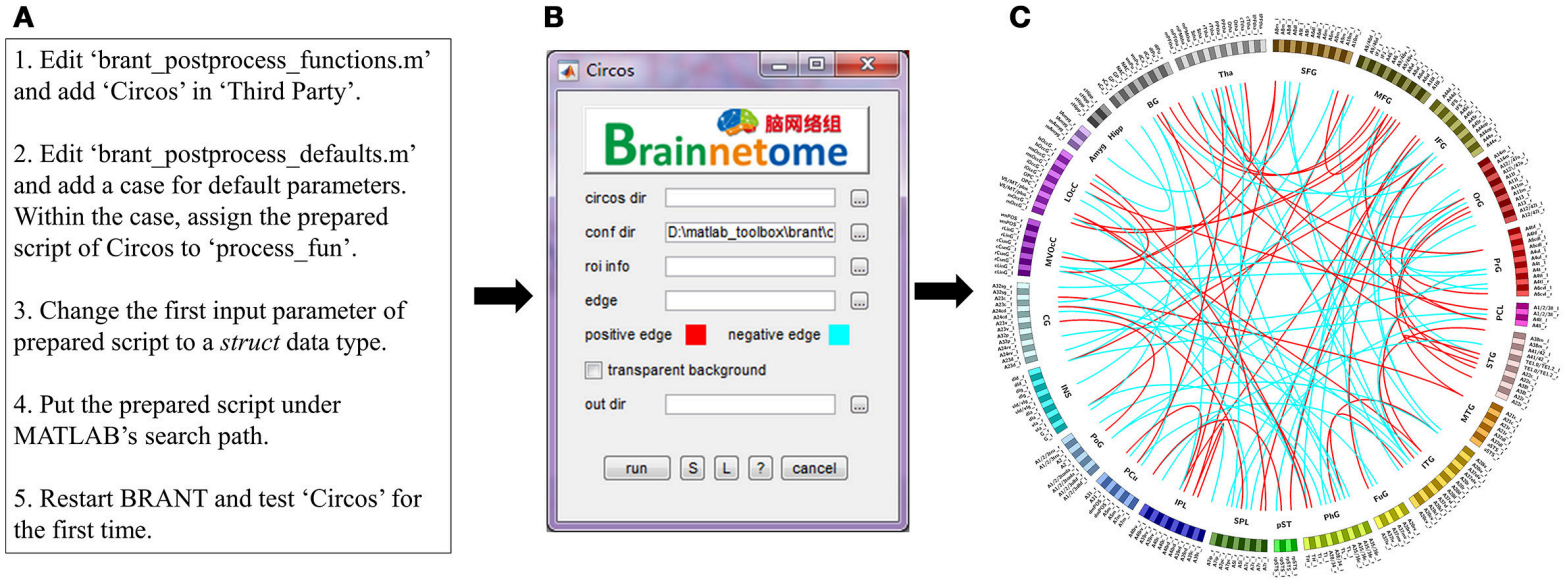
**(A)** An example of adding new functions in BRANT. **(B)** Code-generated input panel of Circos. **(C)** Circos map with randomly generated links for illustration purpose.

Circos (http://circos.ca/) is a Perl-based software designed to visualize pair-wise connections on a ring map (Krzywinski et al., 2009; Irimia et al., 2012; Fan et al., 2016). However, each time users would need to prepare files for *band* and *link*, tune parameters and open a terminal to run commands. In the current function, we use the code-generated GUI to collect input parameters and pass them to a prepared Circos script. Expected input parameters (Figure 3B) include the directory of Circos binaries (*circos dir*), the directory of Circos configuration file (*conf dir*), a descriptive table for ROI information (*roi info*), a file stores the ROI-ROI connectivity matrix (*edges*), colors for positive and negative connectivity (*positive and negative edges*), the choice whether to use transparent background (*transparent background*), and output directory (*out dir*). To construct the Circos input panel, steps in Figure 3A are used.

#### DiffusionKit

DiffusionKit is a diffusion-weighted imaging data processing and fiber-visualizing software compiled with C++ (Xie et al., 2016) (http://diffusion.brainnetome.org). The current version of DiffusionKit is designed to process data one by one with manual input from an input interface, while across-subject batch processing requires terminal scripts. Using similar procedures in Figure 3A, we generated a batch-processing GUI that internally integrates DiffusionKit executives into a pipeline for eddy correction, diffusion tensor imaging (DTI) reconstruction, DTI tracking, high angular resolution diffusion-weighted imaging (HARDI) reconstruction and HARDI tracking.

### Input/output (I/O) optimization

Though metrics in BRANT have been optimized with matrix operations, parallel computing and multithreading, the total computing time is still limited to the speed of file I/O, especially when temporal or spatial resolution of a rs-fMRI image is high. To speed up file I/O, we have written and compiled a MATLAB-C++ mixed ^*^.mex binary with zlib (http://www.zlib.net/) to directly load and save gzipped files without decompression/compression. For illustration purposes, we tested the loading time of a minimally preprocessed (Glasser et al., 2013) subset of Human Connectome Project's working memory data (100 subjects, 405 timepoints, TR 720 ms, https://www.humanconnectome.org/study/hcp-young-adult/data-releases/) on a 7200-rpm Seagate hard drive. As a result, the average loading time of one gzipped file is two times shorter than the uncompressed one (mean ± standard deviation, gzipped: 9.7 ± 1.69 s, uncompressed: 30.18 ± 2.23 s).

### Scripting

When running batch processing on a remote cluster, where GUI operations are not available, we can first save and modify the batch parameters on local machine, then upload the saved parameters to the remote cluster, and run with one command ‘brant_run_script(‘/path/saved_file.mat’)’. The saving operation is tied to the button labeled with “S” (short for “save”) on most GUI panels. Besides, the ^*^.mat files can also be loaded by the button “L” (short for “load”), to initialize panel parameters.

## Compare with MATLAB-based toolboxes

**Figure 4:**
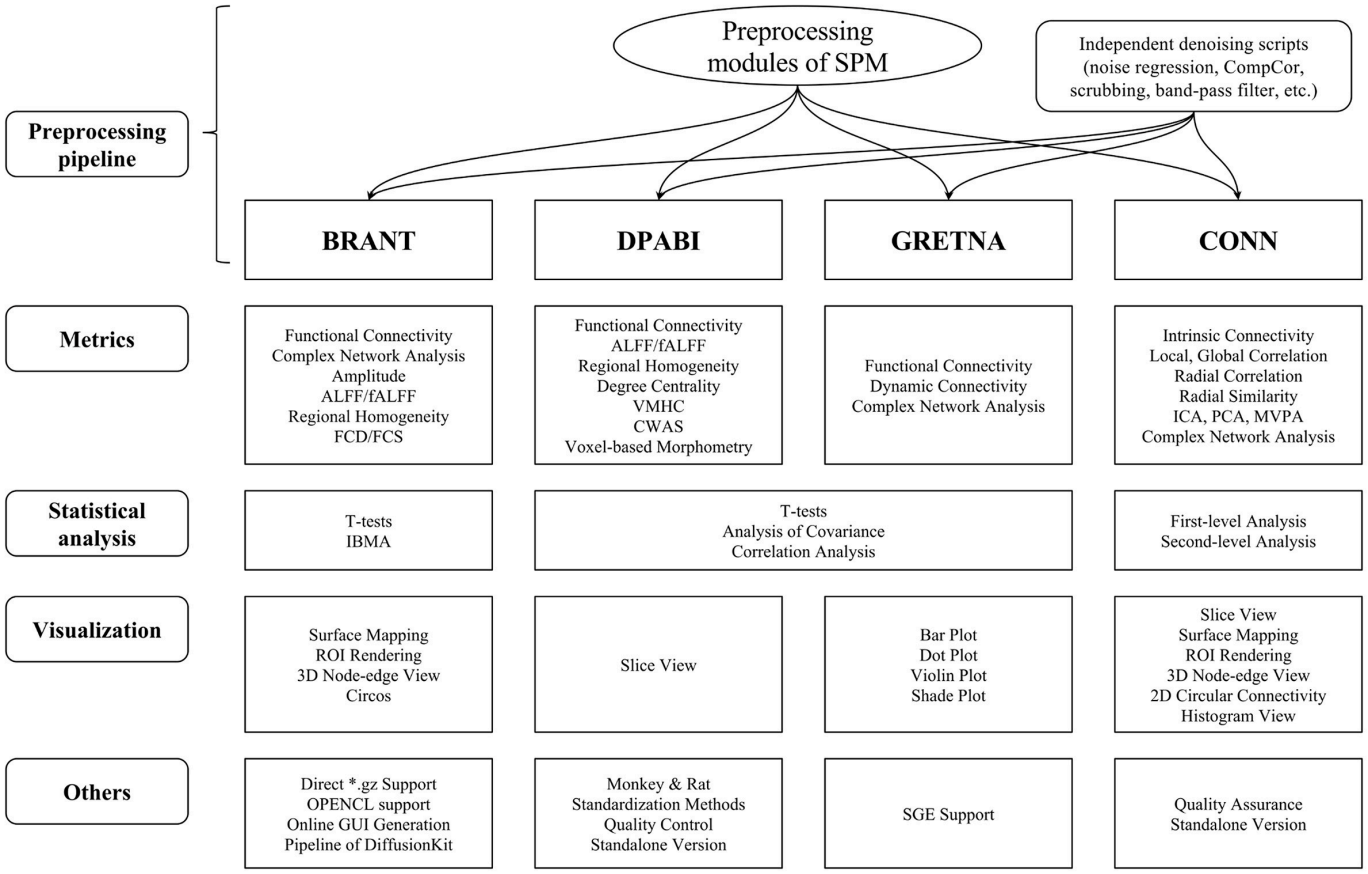
Horizontal comparison of MATLAB-based full-functioning software. CompCor, Component based noise correction method (Behzadi et al., 2007). FCD, Functional connectivity density; FCS, Functional connectivity strength; (f)ALFF, (fractional) Amplitude of low frequency fluctuation; VMHC, Voxel-mirrored homotopic connectivity (Zuo et al., 2010); CWAS, Connectome-wide association studies (Shehzad et al., 2011); ICA, Independent component analysis; PCA, Principle component analysis; MVPA, Multi-voxel pattern analysis; IBMA, Image-based meta-analysis; ROI, Region of interest; SGE, Sun Grid Engine.

The functions of BRANT cover a wide range of data processing, from DICOM conversion to results visualization. Since there exist a number of MATLAB-based toolboxes, we compare BRANT with three other frequently used full-featured toolboxes, DPABI v2.3 (Yan et al., 2016), GRETNA v2.0.0 (Wang et al., 2015), and CONN v17.f (Whitfield-Gabrieli and Nieto-Castanon, 2012) (Figure 4). All the four toolboxes integrated SPM's preprocessing scripts and implemented their own denoising scripts for batch preprocessing. During data preprocessing, a necessary procedure of visually checking raw data quality and normalization quality is implemented in DPABI as *Quality Control*, in CONN as *Quality Assurance* and in BRANT as *Visual Check*. At post-processing, both GRETNA and CONN focus on graph-based analysis and provide a variety of functional connectivity analysis methods and complex network analysis methods. DPABI provides functions of voxel-based methods (e.g. ALFF, ReHo, voxel-mirrored homotopic connectivity Zuo et al., 2010) and functional connectivity analysis. In BRANT, functional connectivity analysis, complex network analysis and several voxel-based metrics of spontaneous activity are implemented. To test statistical hypotheses, BRANT implemented the Student's *t*-test for second-level analysis and the IBMA for element-wise meta-analysis, but neither the analysis of covariance in DPABI and GRETNA, nor the subject-level and group-level analysis in CONN. For results visualization, CONN provides a wide range of methods to plot voxel-wise connectivity in histogram, to overlay voxel intensity on slices and surfaces, to render ROIs in 3D clusters, and to plot connectivity in 2D circular and 3D node-edge view. In DPABI, slice viewer is provided with thresholding and atlas labeling options, and in GRETNA, bar plot, dot plot, violin plot, and shade plot are implemented. In BRANT, results visualization functions include surface mapping for voxel intensity, ROI rendering, 3D node-edge view (Figure 2), and Circos 2D circular view (Figure 3). Besides data processing, statistical analysis, quality control and visualization methods, CONN and DPABI have compiled standalone versions, which can run without MATLAB. Moreover, DPABI provides pipelines tuned for monkey and rat data processing. In GRETNA, Sun Grid Engine (SGE) support is available for submitting data processing jobs to a remote computer cluster. In BRANT, we have added direct ^*^.gz support for most post-processing functions, OPENCL-based parallel computing for time consuming FCD/FCS calculation and online GUI generation for almost all functions.

In the comparison, functions of GRETNA and CONN focus on a variety of metrics for functional connectivity analysis and complex network analysis, while DPABI and BRANT provide a wider range of data analysis that cover not only connectivity-based and graph-based analyses, but also voxel-based spontaneous activity analysis. As for GUI design, both DPABI and GRETNA use manually drawn figures as input panels, while BRANT automatically generates GUIs by extendable scripts.

## Examples of rs-fMRI data analysis

To validate the efficacy of our toolkit, we used the same preprocessing pipelines provided by BRANT and DPABI v2.3, to compare ReHo, fALFF and FCs using a rs-fMRI dataset consists of 18 patients with mild cognitive impairment (MCI), 17 patients with mild Alzheimer's disease (mAD), 18 patients with severe Alzheimer's disease (sAD) and 21 normal controls (NC). This dataset was used in our previous study (Liu et al., 2014) and made available online at https://github.com/yongliulab.

### Subjects, data acquisition, and processing

The details of the dataset can be found in our previous studies (He et al., 2014; Liu et al., 2014, 2016). Therefore, in the current paper we provide a brief introduction about the subjects' inclusion and exclusion criteria, data acquisition and processing to maintain the scientific integrity of the present study. All the participants were recruited by advertisement and supported throughout the testing procedures in a specialist neuropsychological research facility at Xuanwu Hospital, Beijing, China. Patients and informants (usually a family member) were clinically interviewed by a senior neurologist. Written consent forms were obtained from all subjects or their legal guardians (usually a family member). The study was approved by the ethics committee of Xuanwu Hospital. AD subjects were diagnosed using standard operationalized criteria (Diagnostic and Statistical Manual of Mental Disorders, Fourth Edition (DSM-IV); American Psychiatric Association 1994 and National Institute of Neurological and Communicative Disorders and Stroke - Alzheimer's Disease and Related Disorders Association (NINCDS-ADRDA); McKhann et al., 1984). The severity of dementia was assessed using the Clinical Dementia Rating (CDR) scale (Morris, 1993). Patients with a diagnosis of AD and CDR score of 1 were classified as mild AD and those with a CDR score of 2 or 3 were diagnosed as severe AD. MCI was diagnosed according to standard criteria (Petersen et al., 1999, 2001; Choo et al., 2007), which included subjective memory loss with objective evidence of memory impairment in the context of normal or near-normal performance on other domains of cognitive functioning; minimal impairment of activities of daily living; and a CDR score of 0.5. Normal volunteers have a CDR score of 0. All participants satisfied the following inclusion criteria: (1) no history of an affective disorder within 1 month prior to assessment; (2) normal vision and audition; (3) able to cooperate with cognitive testing; (4) aged between 50 and 90 years; (5) no clinical history of stroke or other severe cerebrovascular disease; and (6) no more than one lacunar infarction, without patchy or diffuse leukoaraiosis, on neuroradiological assessment of conventional MR images. The exclusion criteria included: (1) severe general medical disorders of cardiovascular, endocrine, renal, or hepatic systems; neurological disorders associated with potential cognitive dysfunction, including local brain lesions, traumatic brain injury with loss of consciousness or confusion, and dementia associated with neurosyphilis, Parkinsonism, or Lewy body disease; psychiatric disorders including depression, alcohol, or drug abuse; (2) concomitant use of psychotropic medication in large quantity; and (3) insufficient cognitive capacity to understand and cooperate with study procedures. All patients underwent a complete physical and neurological examination, an extensive battery of neuropsychological assessments, and standard laboratory tests. Healthy volunteers underwent a brief clinical interview and MMSE to confirm that they satisfied exclusion criteria for cognitive deficits, psychoactive drug use, and clinical disorders.

The MR images were acquired on a 3.0-T MR scanner (Magnetom Trio, Siemens, Germany). Functional MRI data were acquired using an echo planar imaging (EPI) sequence sensitive to BOLD contrast: Repetition time (TR) = 2,000 ms, echo time (TE) = 30 ms, flip angle (FA) = 90°, matrix = 64 × 64, field of view (FOV) = 220 mm × 220 mm, slice thickness = 3 mm with inter-slice gap = 1 mm. Each brain volume comprised 32 axial slices, and each scanning session lasted for 360 s. Sagittal T1-weighted MR images were acquired by a magnetization-prepared rapid gradient-echo sequence (TR/TE = 2000/2.6 ms, FA = 9°, matrix = 256 × 224, FOV = 256 mm × 224 mm, 176 continuous sagittal slices with 1 mm thickness).

To compare the results between different toolkits, we performed the same processing pipeline in the present study. After DICOM to NIFTI conversion, the first 10 timepoints of EPI images were discarded. The following consecutive rs-fMRI timepoints were processed with the BRANT pipeline, which includes slice timing correction, within subject registration, rigid-body registration of T1 image to EPI mean image, normalization of EPI images to MNI standard space using T1 image. After normalization, EPI images were resampled to 3 × 3 × 3 mm^3^. The co-registration and spatial normalization functions integrated in BRANT, DPABI and GRETNA are slightly different. For example, prior to T1 to rs-fMRI co-registration, BRANT firstly segments the T1 image (with SPM-*Segment*) to tissue probability maps in individual space, and then extract the brain with gray matter probability + white matter (WM) probability + cerebrospinal fluid (CSF) probability > 0.5 as mask, while DPABI uses *bet* with tuned parameters from FSL to extract brain, and in GRETNA, no brain extraction method was found. To compare all software with same preprocessing procedures, we used the spatially normalized results processed with BRANT and ran same denoising processes in BRANT, DPABI and GRETNA for the following calculations and comparisons. The denoising process consists of linear and quadratic trends removal, covariates regression and band-pass filtering within 0.01-0.08 Hz. Covariates include mean and squared signals of WM and CSF (extracted by same masks across different software), and the Friston's 24-parameter head motion model (Friston et al., 1996). To mask out spurious voxels in the gray matter, we used the intersection between group mean TSNR mask (intensity > 30) and a gray matter mask (probability >30%), to mask the following fALFF and ReHo calculation, as well as the AAL atlas used for ROI-wise FC calculation. We calculated fALFF within frequency band 0.01~0.08 Hz with the unfiltered images, while we calculated ReHo (with 27 neighboring voxels) and FCs with the preprocessed images. The ReHo and fALFF maps were normalized by z-score transform and then smoothed with a 6 × 6 × 6 mm^3^ Gaussian kernel, while the FCs were Fisher-z transformed. We performed the *t*-tests, with age, education and gender regressed out as covariates, to compare the group mean differences of FCs (among BRANT, GRETNA, and DPABI), fALFF (between BRANT, and DPABI) and ReHo (between BRANT, and DPABI).

## Results

**Figure 5:**
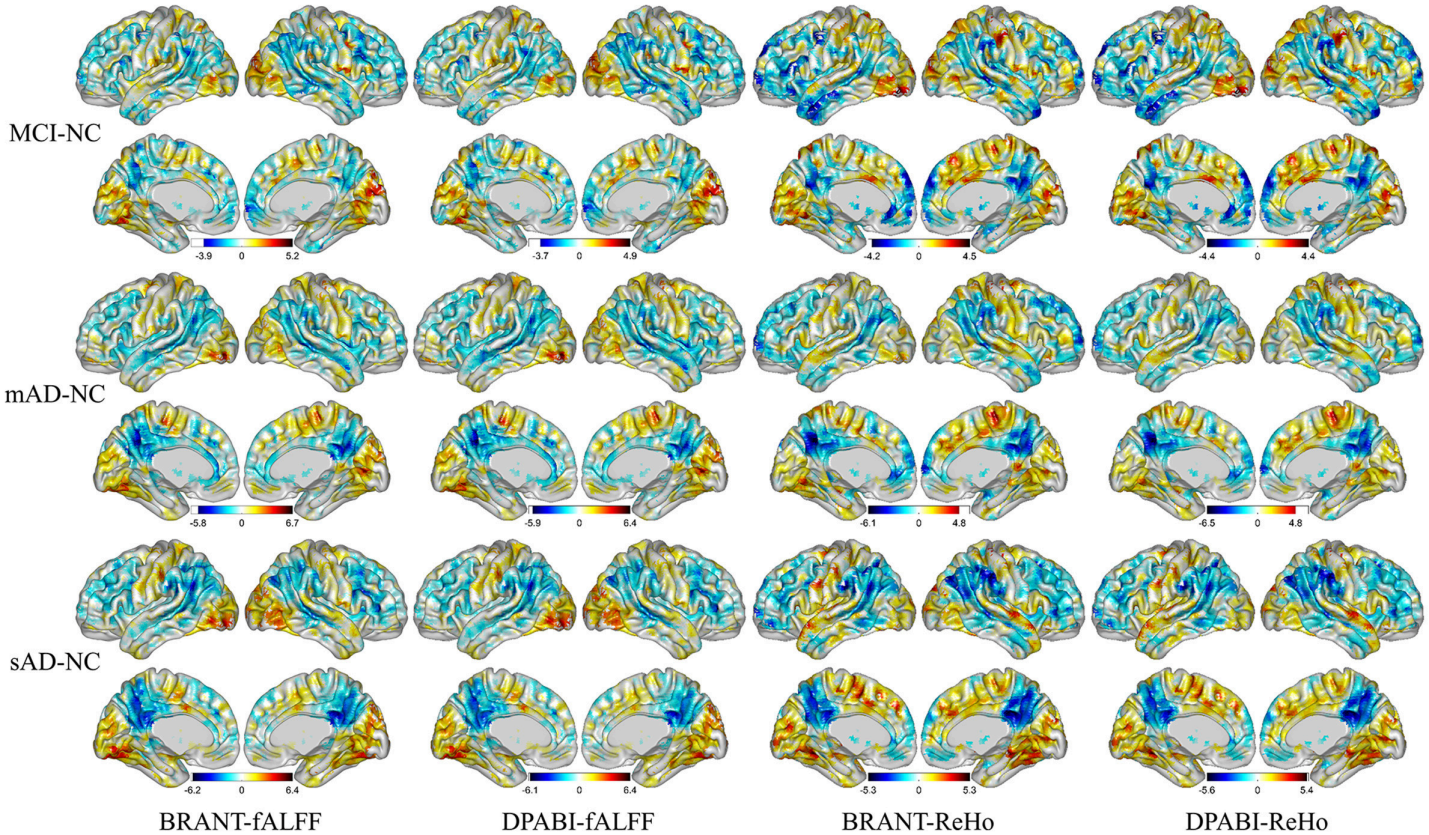
T-statistic maps of ReHo and fALFF. The most severely decreased fALFF and ReHo in patient groups were found at posterior cingulate cortex, precuneus, supramarginal gyrus, angular gyrus, inferior parietal lobule. Increased ReHo and fALFF in patient groups were mainly found at gyrus at medial and inferior occipital lobule.

**Figure 6:**
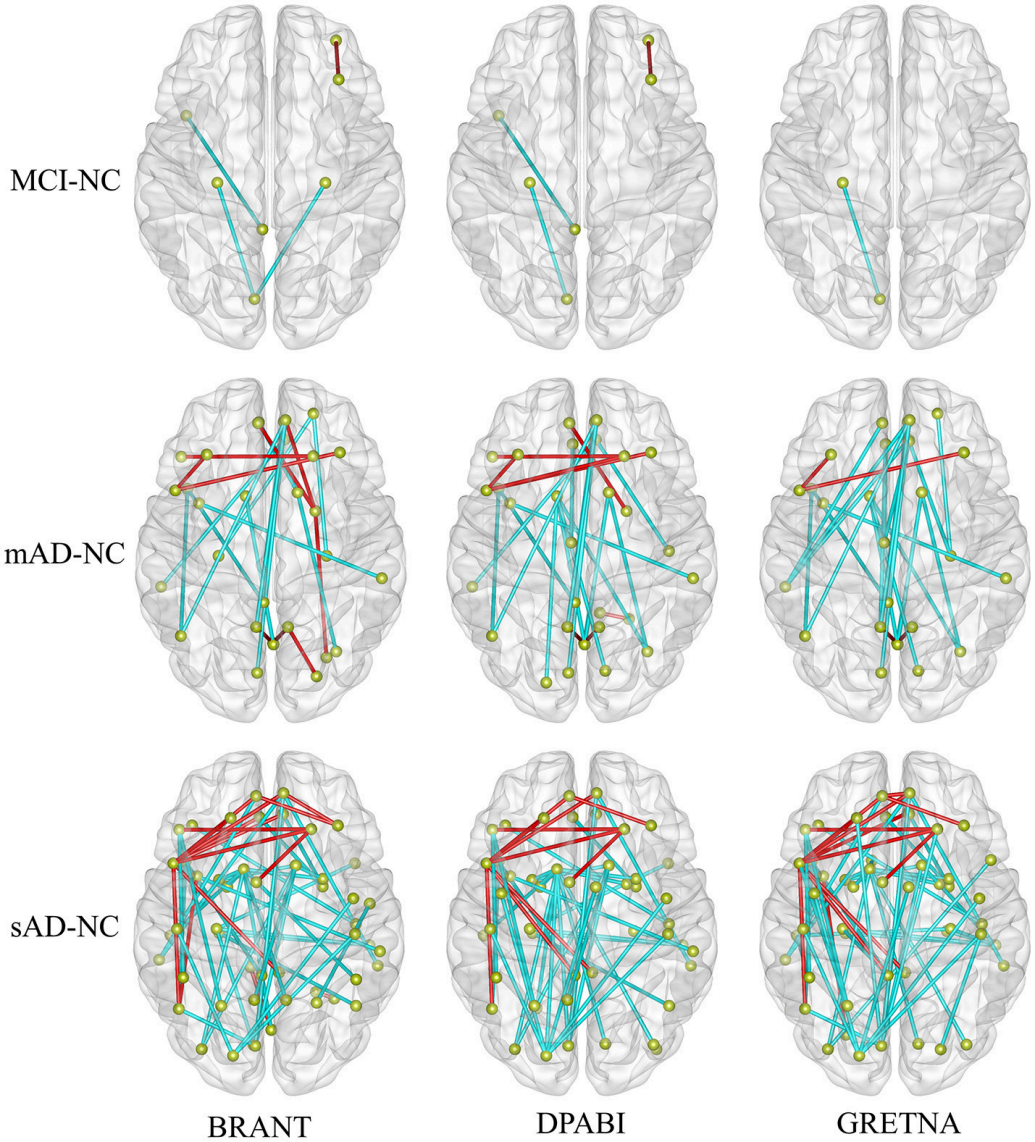
The altered functional connectivity drawn within brain surfaces (*P* < 0.001, uncorrected). As shown in the figure, more abnormal FCs were found with the development of Alzheimer's disease. In the contrast of sAD and NC groups, decreased FCs (in cyan) were mainly found between medial frontal areas and bilateral middle temporal gyrus, between medial frontal areas and posterior cingulate gyrus, between bilateral caudate and occipital areas, between superior occipital cortex and bilateral pre-/postcentral gyrus, while increased FCs (in red) were mainly found between the left inferior frontal and the medial and dorsolateral frontal areas.

No significant differences (*P* > 0.05) of age (two-tailed two sample *t*-test), gender (chi-squared test) and education (two-tailed two sample *t*-test) were found between each patient group and NC group. T-statistic maps of fALFF, ReHo (Figure 5) and results of FCs (*P* < 0.001, uncorrected) (Figure 6) based on different toolkits have quite similar patterns, which suggest BRANT is another optimal toolkit for rs-fMRI research community. In the results, the minor differences can be induced by different implementations of trends removal, covariance regression and band-pass filter. For example, in trends removal and covariates regression steps, BRANT and DPABI put the temporal trends within the regression model, while in GRETNA trends removal and covariates regression are two separate processes. In band-pass filtering, BRANT periodically extends time series with mirrored ones, while DPABI and GRETNA pad time series with zeroes. For the results, we didn't draw a strong conclusion, since the interpretation requires multiple comparison corrections and is not the main point of the current study. Moreover, there exist other algorithms which can better remove the motion related signals as discussed in one recent study (Ciric et al., 2017), such as timepoints scrubbing (Power et al., 2012, 2014), and component based noise correction methods (Behzadi et al., 2007; Pruim et al., 2015a,2015b).

## Summary of the BRANT

We presented the construction and the key features of BRANT in the manuscript and put online the updated instructions and implementation details (http://brant.brainnetome.org for instructions and stable BRANT versions), to keep the toolbox updated and compatible to newer SPM and MATLAB versions.

Among the above rs-fMRI data processing toolboxes, our package has two distinguishing advantages. One is, other than focusing on several specific types of rs-fMRI data processing, functions of BRANT cover a wide range of rs-fMRI data processing methods. The other advantage lies in the implementation process that GUIs are created automatically with a few lines of MATLAB code instead of drawn manually. On the other side, BRANT also has some limitations in several modules. For example, in denoising pipeline, we have added regression models composed of tissue mean signals, and head motion estimates in the regression model, but not the independent component analysis (ICA) based methods such as the ICA-AROMA method (Pruim et al., 2015a,b), which was found better at removing distance-dependent motion artifacts (Ciric et al., 2017). In statistical analysis, we have implemented Student's *t*-tests for group mean comparisons, but the first-level analysis for activation detection, and the analysis of variance for multi-group variances inference are yet not implemented and will be added in the future. In network analysis, a number of thresholds and random networks are induced and the computation is very intense even running with parallel workers on single machine. To reduce the computing time, the pipeline system for Octave and Matlab (PSOM) (Bellec et al., 2012) can be used to submit the jobs in parallel to a computer cluster.

In summary, we have developed a GUI-based MATLAB toolbox, which consists of modules for preprocessing, voxel-based spontaneous activity analysis, functional connectivity analysis, complex network analysis, statistical analysis, and results visualization. In the toolbox, scripts are optimized by efficient file handling methods and parallel computing, while functions are made easily extendable by code-generated GUIs. Along with the contributions from open source community, we seek to provide more robust and versatile versions in the future.

## Dependencies

Part of BRANT's functions depends on existing packages or executives. All preprocessing steps (except for *Denoise*), file and folder I/O dialogue boxes and *VISUAL CHECK*, use functions of SPM (Ashburner, 2012). The conversion of DICOM to NIfTI uses *dcm2nii* from MRIcron, an updated version of Mricro (Rorden and Brett, 2000). Most NIfTI file loading and saving scripts use scripts of *Tools for NIfTI and ANALYZE image* (http://cn.mathworks.com/matlabcentral/fileexchange/8797-tools-for-nifti-and-analyze-image). The scroll bars (e.g., in the parameter panel of *Preprocessing*) are provided by *FINDJOBJ* (http://cn.mathworks.com/matlabcentral/fileexchange/14317-findjobj-find-java-handles-of-matlab-graphic-objects).

## Author contributions

TJ and YL designed the project. KX, YL, YZ, and JR contributed to the source code. KX and YL wrote the paper.

### Conflict of interest statement

The authors declare that the research was conducted in the absence of any commercial or financial relationships that could be construed as a potential conflict of interest.
